# Integrated Multimodal Imaging for Transvenous Transpulmonary Atrial Pacing Lead Implantation in Fontan Patient

**DOI:** 10.1016/j.jaccas.2025.105286

**Published:** 2025-10-01

**Authors:** Miranda J. Flores, Lauren Sell, Anjan S. Batra, Sanjay P. Sinha, Gira S. Morchi, Joanne P. Starr, Anthony C. McCanta

**Affiliations:** a Children's Hospital Orange County, Orange, California, USA; b University of California–Irvine, Irvine, California, USA; c University of California–Los Angeles, Los Angeles, California, USA

**Keywords:** Fontan, intracardiac echocardiography, pacemaker, protein-losing enteropathy, three-dimensional mapping

## Abstract

**Background:**

Pacemaker placement in Fontan patients is challenging owing to lack of venous continuity with the atrial and ventricular myocardium. The standard epicardial approach is complicated by the need for repeat sternotomy or thoracotomy as well as the overall long-term risk of lead failure. Transvenous lead implant has been described, but this requires transpulmonary or transbaffle puncture, which have risks of bleeding and pericardial effusion.

**Case Summary:**

We describe a case in which multimodal imaging with fluoroscopy, computed tomography, intracardiac echocardiography, and three-dimensional electroanatomical mapping were combined to perform successful transvenous transpulmonary epicardial atrial lead placement in an extracardiac Fontan patient with protein-losing enteropathy.

**Discussion:**

This approach can be considered as an alternative to epicardial lead placement in Fontan patients who are at moderate to high risk with the standard epicardial approach.

**Take-Home Messages:**

Congenital heart patients with extracardiac Fontan repairs pose unique technical challenges to pacemaker implantation given the lack of venous continuity with the myocardium. The transvenous, transpulmonary approach to epicardial atrial lead placement can be considered in high-risk extracardiac Fontan patients, using integrated multimodal imaging techniques to maximize safety and success.

## Graphical Abstract

**Figure:**
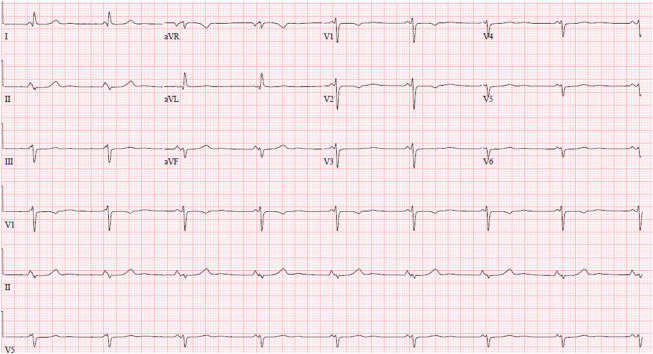

## History of Presentation

The patient was a 14-year-old male adolescent with congenital heart disease who presented with near-syncope and a fall while walking at school, with associated visual aura, fatigue, and emesis. After 2 hours of significant fatigue and “lightheadedness,” he was taken to the emergency department, where he was found to be alert and oriented with no respiratory distress and with pulse of 67 beats/min, blood pressure of 108/57 mm Hg, and oxygen saturation level of 98%. His heart rate was regular with single S2, lungs were clear bilaterally, and abdomen was soft and nontender, but with mild diffuse ascites and mild bilateral lower extremity pitting edema.

**Take-Home Messages**

• Congenital heart patients with extracardiac Fontan repairs pose unique technical challenges to pacemaker implantation given the lack of venous continuity with the myocardium.
• The transvenous, transpulmonary approach to epicardial atrial lead placement can be considered in high-risk extracardiac Fontan patients, using integrated multimodal imaging techniques to maximize safety and success.

## Past Medical History

The patient had a history of complex single-ventricle physiology, including double outlet right ventricle with d-transposition of the great arteries and large inlet ventricular septal defect. After initial palliations with a central shunt at 1 month of age and right bidirectional Glenn shunt at 8 months, he underwent a 16-mm extracardiac Fontan procedure with tricuspid valve oversewing and dual-chamber epicardial pacemaker implantation for sinus node dysfunction at 3 years of age. His atrial pacing lead failed at age 7 years, and he was changed to VVIR pacing mode. He had not returned to scheduled follow-up visits or sent remote pacemaker interrogations for 2 years prior to the current presentation.

## Differential Diagnosis

The differential diagnosis included symptomatic bradycardia due to sinus node dysfunction with pacemaker malfunction with junctional bradycardia. Paroxysmal tachycardia including intra-atrial re-entrant tachycardia, ectopic atrial tachycardia, and ventricular tachycardia were also considered. Primary systolic or diastolic single ventricle dysfunction was in the differential diagnosis. Protein-losing enteropathy (PLE) due to Fontan circulation failure with elevated pulmonary vascular resistance was also considered.

## Investigations

**Figure 1:**
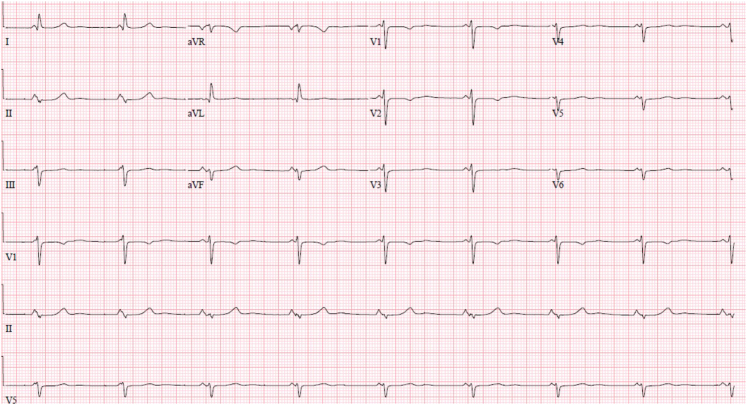
12-Lead Electrocardiogram at Presentation Electrocardiogram demonstrating junctional bradycardia competing with sinus bradycardia and nonspecific ST-segment abnormality.

**Figure 2:**
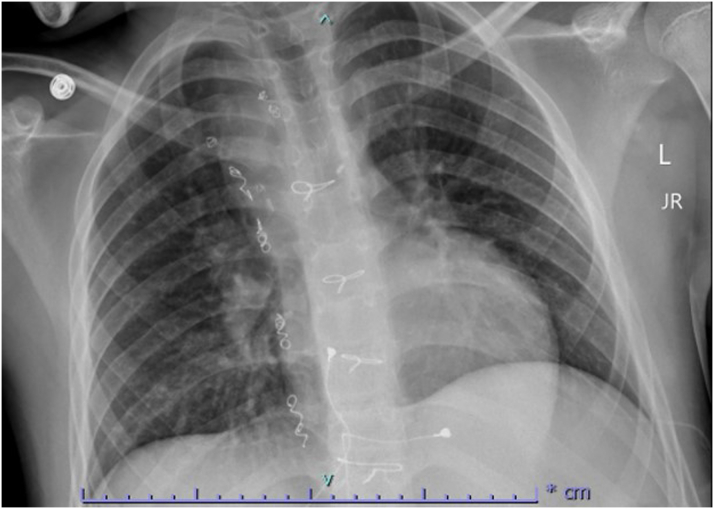
Chest X-Ray at Presentation Chest x-ray with no cardiomegaly, no pulmonary congestion, and with epicardial pacing wires attaching to the atrial and ventricular epicardium and sternal wires from prior sternotomies overlying the central chest.

Initial electrocardiogram (Figure 1) showed junctional rhythm with ventricular rate of 67 beats/min, no evidence of atrial activity, and no evidence of pacemaker activity. Pacemaker interrogation revealed ventricular lead impedance >3,000 Ω without sensing or capture and with battery at elective replacement. A chest x-ray (Figure 2) showed clear lungs, no cardiomegaly, and epicardial pacing leads without evidence of fracture. Transthoracic echocardiogram demonstrated normal single left ventricle systolic function, moderately dilated left atrium, no Fontan fenestration, no significant mitral valve regurgitation, and phasic flow in the Glenn and Fontan shunts.

Laboratory studies revealed normal hemoglobin and renal function. Albumin was low (2.4 g/dL), as well as total protein (4.4 g/dL), immunoglobulin G (228 g/dL), and immunoglobulin M (44 g/dL). Stool alpha-1-antitrypsin was elevated (595 mg/dL).

A computed tomography angiogram (CTA) was performed, which confirmed patent pulmonary arteries and Fontan, patent atrial septectomy, and patent aortic arch, and which delineated the relationship of the left pulmonary artery floor and the roof of the common atrium.

**Figure 3:**
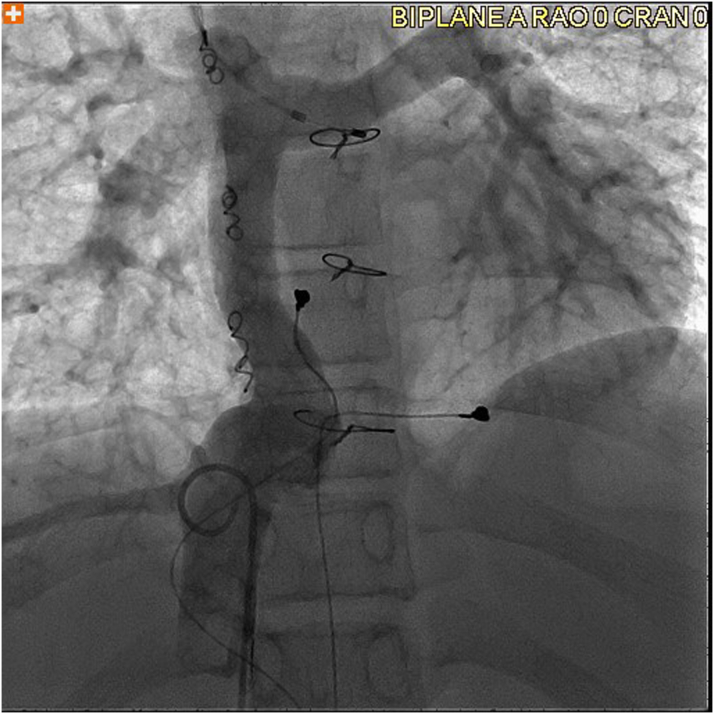
Angiography of Fontan Baffle and Left Pulmonary Artery Venogram from a pigtail catheter just below the hepatic vein to Fontan junction showing patent extracardiac Fontan with no evidence of distal Fontan obstruction and patent left pulmonary artery. A temporary pacing catheter is placed from the right internal jugular vein at a location with atrial pacing capture.

The patient underwent cardiac catheterization, which was significant for systemic oxygen saturation of 89%, mean Fontan and pulmonary artery pressure of 12 mm Hg, and left ventricular end-diastolic pressure of 7 mm Hg. As baseline hemodynamics were performed in junctional rhythm, limited hemodynamics were also performed with temporary atrial pacing from the pulmonary artery floor, capturing the atrial roof at 90 beats/min (Figure 3). With atrial pacing, Fontan and pulmonary artery pressures decreased to a mean of 10 mm Hg for the Fontan/pulmonary arteries. A transjugular liver biopsy was performed and confirmed congestive hepatopathy, with a fibrosis score of 1 and mild pericentral vein and pericellular fibrosis without bridging and with mild sinusoidal congestion. Venovenous collaterals were occluded.

## Management

The patient was diagnosed with ventricular lead failure and PLE thought to be due to low cardiac output secondary to sinus node dysfunction and junctional escape rhythm. Milrinone was started to improve cardiac output. Permanent pacemaker replacement with the placement of an atrial pacing lead was recommended. The case was discussed at a comprehensive congenital cardiac surgical conference, but because the risks of repeat sternotomy and/or thoracotomy in this patient with PLE were felt to be high, the cardiac surgical team requested attempt at the transvenous, transpulmonary approach.

**Figure 4:**
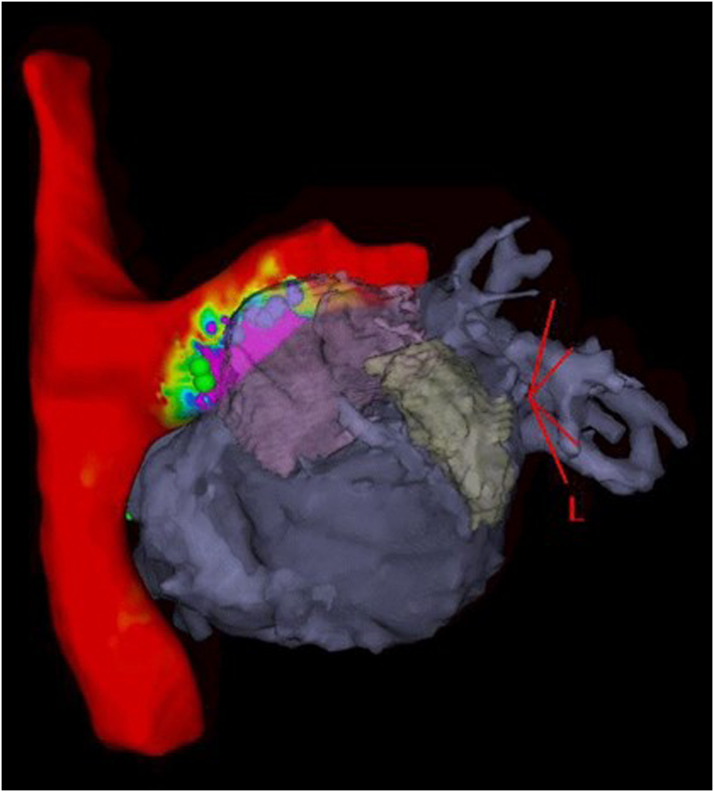
3-Dimensional Electroanatomical Map of the Left Pulmonary Artery Combined With Computed Tomography Angiogram of the Left Atrium and Right Atrium Electroanatomic voltage map of the superior vena cava, Fontan baffle, and left pulmonary artery with three-dimensional reconstruction of the common atrium with left atrial appendage and pulmonary veins showing the proximity of the atrium underneath the left pulmonary artery. Voltage map with color gradient: red = voltage <0.1 mV; purple = voltage >0.5 mV.

Prior to the procedure, the CTA was integrated into a three-dimensional (3D) electroanatomical mapping system. Access was obtained to the bilateral femoral veins and the right internal jugular vein. An 8-F intracardiac echocardiography (ICE) catheter was advanced from the left femoral vein to the Fontan conduit and bifurcation of the pulmonary arteries, and 3D geometry of the Fontan, atrium, and left pulmonary artery was obtained and integrated with the CTA on the 3D mapping system. Then, a 20-pole mapping catheter was advanced from the right femoral vein, and a comprehensive voltage map of atrial electrical activity from the left pulmonary artery was added to the existing 3D geometry (Figure 4).

The right internal jugular sheath was exchanged over a 0.014-inch wire for a deflectable delivery catheter, and this was advanced to the left pulmonary artery adjacent to sites of voltage seen on the 3D mapping system. A 71-cm trans-septal needle was advanced over the 0.014-inch wire to avoid puncture of the delivery catheter.

**Figure 5:**
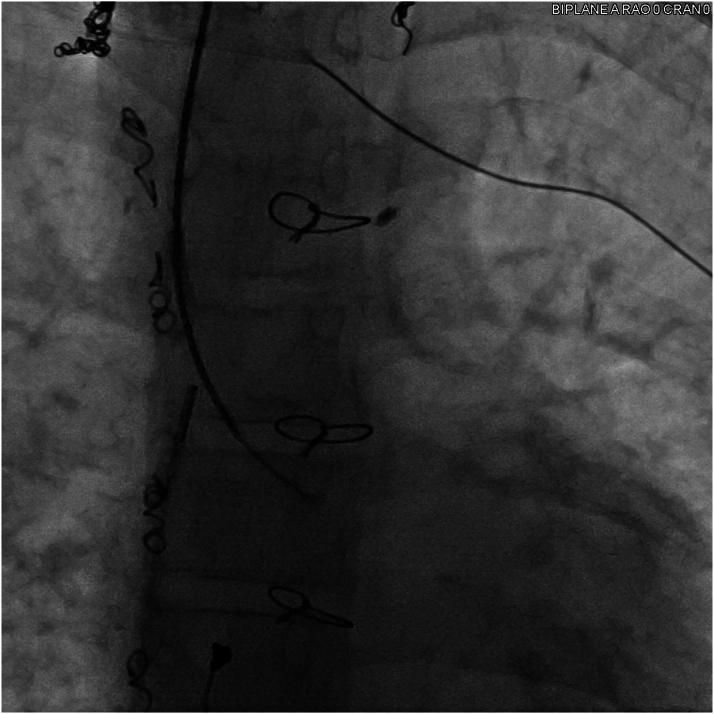
Fluoroscopy of Transpulmonary Puncture Trans-septal needle from the superior vena cava puncturing through the proximal left pulmonary artery to the epicardial space. The intracardiac echocardiography catheter is positioned from the right femoral vein across the Fontan baffle at the bifurcation of the pulmonary arteries, with the ultrasound vector directed at the trans-septal needle and transpulmonary puncture site.

**Figure 6:**
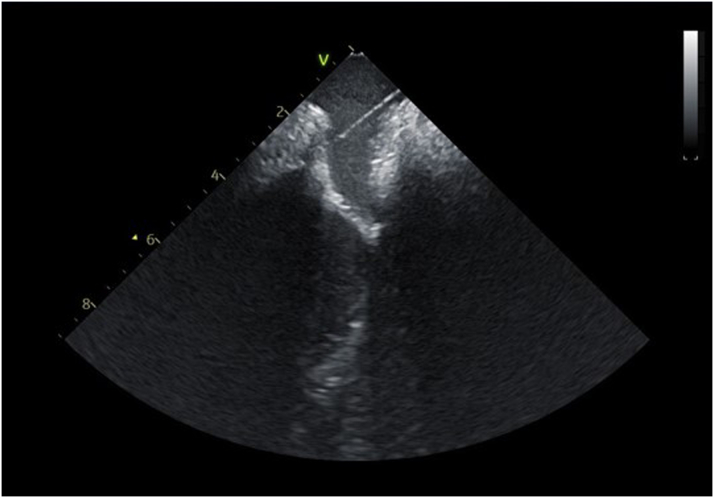
Intracardiac Echocardiography of Transpulmonary Puncture Intracardiac echocardiography image of trans-septal needle extending from the superior vena cava (rightward) into the floor of the proximal left pulmonary artery directed toward the epicardial space.

**Figure 7:**
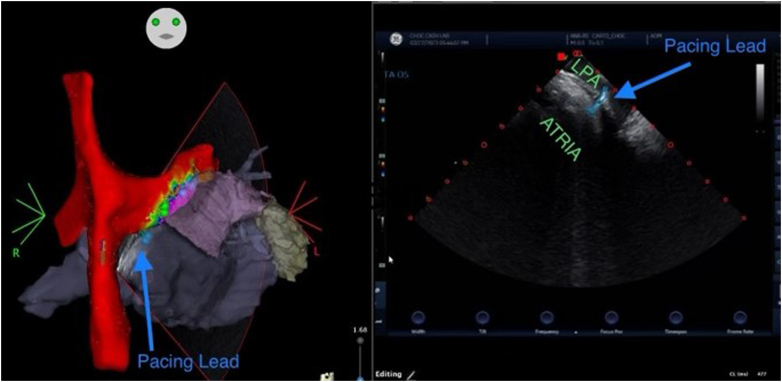
Integrated 3-Dimensional Image of Final Lead Placement (Left Panel) Multimodal three-dimensional image combining electroanatomical voltage map, computed tomography angiogram, and intracardiac echocardiography catheter location and ultrasound sector projected onto the map, with pacing lead outlined in light blue. Voltage map with color gradient: red = voltage <0.1 mV; purple = voltage >0.5 mV. (Right Panel) Intracardiac echocardiography image of pacing lead transversing the left pulmonary artery and affixed to the epicardial common atrium. LPA = left pulmonary artery.

Using the combined CTA, ICE, and 3D map to target a location with underlying voltage, transpulmonary puncture was performed under fluoroscopic and ICE guidance (Figures 5 and 6). Of note, while the voltage map indicated higher voltage more laterally underlying the left pulmonary artery, transpulmonary puncture was not possible in that location owing to a mismatch of the acute angle of the left pulmonary artery and the curve of the trans-septal needle, resulting in inability to transmit sufficient forward pressure to puncture the pulmonary artery floor. After transpulmonary puncture, the delivery catheter was advanced over the needle into the epicardial space, and the needle was removed. The pacing lead was advanced through the delivery catheter, and brief mapping of the underlying myocardium was performed, although once the delivery catheter was in the epicardial space, only subtle movements of <1 cm were possible. The lead was affixed to the atrial myocardium at a site with pacing threshold 1.5 V at 0.4 milliseconds, impedance 890 Ω, and P-wave amplitude 2.7 mV. The pacing lead adherence to the epicardial atrial wall was evaluated by ICE, and the integrated ICE, CTA, and 3D map confirmed the lead location (Figure 7, Videos 1 and 2). The delivery catheter was split and removed under ICE and fluoroscopic guidance, confirming no pericardial effusion. After lead slack was added to account for patient growth, the lead was secured to the subcutaneous fascia adjacent to the internal jugular vein and tunneled to an infraclavicular prepectoral pocket on the right chest, where it was connected to a new generator. The pacemaker was programmed to AAI pacing mode with low rate (70 beats/min).

## Outcome and Follow-Up

**Figure 8:**
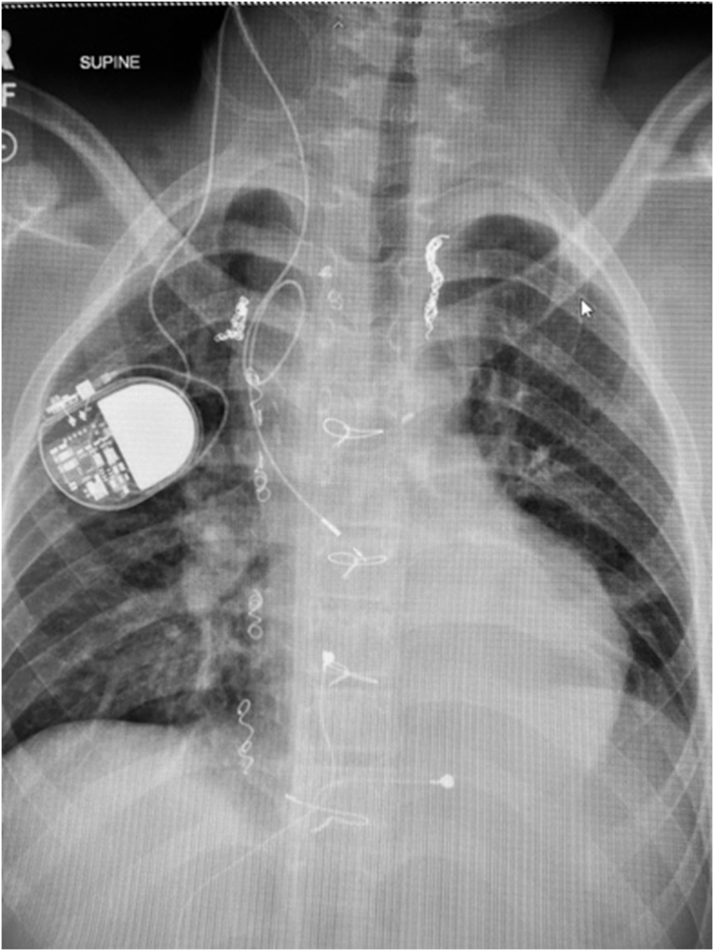
Final Chest X-Ray Anterior-posterior chest x-ray demonstrates transvenous transpulmonary atrial pacing lead placed through the right internal jugular vein and superior vena cava onto the epicardial common atrium with generator overlying right upper chest.

Within 24 hours postprocedure, the pacing threshold improved to 0.75 V at 0.4 milliseconds, and the pacing system appeared stable on chest x-ray (Figure 8). Anticoagulation with rivaroxaban was started, and the patient was weaned off milrinone and discharged home within 3 days. At the 3-month follow-up of AAI pacing at rate 70 beats/min, PLE had resolved, with normalization of albumin, total protein, and immunoglobulins and no evidence of alpha-1-atnitrypsin in his stool. Further, there was no evidence of thromboembolic complications or complications of system anticoagulation. At the 1-year follow-up, the patient remained asymptomatic, with pacing threshold 0.75 V at 0.4 milliseconds and estimated battery life >10 years.

## Discussion

This case describes a patient who had developed PLE owing to low cardiac output from bradycardia and lack of atrioventricular synchrony after ventricular pacing lead failure. Epicardial pacemaker placement, either via sternotomy or thoracotomy, is the standard approach in Fontan patients because Fontan circulation does not readily allow for a leadless or transvenous approach to pacemaker placement given the lack of venous continuity to the myocardium.^1^ However, epicardial pacing has been associated with higher procedural risk, pacing lead failure, and clinical deterioration postoperatively,^2^, ^3^, ^4^ and the cardiac surgical team determined that the risk in our patient was not insignificant. As such, alternative means of pacing were considered.

Although transvenous epicardial atrial lead placement through a transpulmonary puncture has been successfully performed in patients at high risk for epicardial lead placement, published cases have primarily used fluoroscopic imaging.^5^, ^6^, ^7^, ^8^ Because of the potential risks of transpulmonary epicardial lead placement in our patient, integrated multimodal imaging combining fluoroscopy and angiography with CTA, ICE, and 3D electroanatomical mapping was used to perform targeted transpulmonary puncture under direct visualization to minimize risk while optimizing lead placement. In addition, by positioning the lead in a location with low pacing threshold (0.75 V and 0.4 milliseconds), long-term pacing with excellent pacemaker longevity was possible. By restoring atrioventricular synchrony in this Fontan patient, resolution of PLE was observed within 3 months after implant, although long-term lead performance compared with a standard epicardial approach remains unclear.

## Conclusions

Integrated multimodal imaging allowed for safe and targeted transvenous transpulmonary atrial lead implantation in a patient with congenital heart disease and an extracardiac Fontan repair. Using this approach, PLE was successfully treated with minimal procedural burden. By combining all available imaging and electrophysiological techniques, this approach can be considered as a viable and effective alternative to epicardial pacing in high-risk extracardiac Fontan patients.

**Equipment List:** 

3D electroanatomical mapping system	• CARTO 3 (Biosense Webster) including integration modules: • CARTO-MERGE, integration of CTA with 3D map • CARTO-SOUND, integration of intracardiac echocardiography with 3D map
Intracardiac echocardiography	• 8-F Soundstar (Biosense Webster)
Mapping catheter	• 8-F Penta-Ray deflectable 20-pole (Biosense Webster)
Trans-septal needle	• Brockenbrough BRK1 71-cm (Abbott)
Guidewire	• 0.014-inch Transend, M003468150 (Stryker)
Pacemaker delivery catheter	• 9-F SelectSite, C304, L69 catheter (Medtronic)
Pacemaker lead	• SelectSecure, 3830, 69-cm lead (Medtronic)
Pacemaker generator	• Attesta SR MRI, ATSR01 (Medtronic)

3D = 3-dimensional; CTA = computed tomography angiogram.

## Funding Support and Author Disclosures

Dr Batra has been a consultant and member of the speakers bureau with Biosense Webster/Johnson & Johnson. Dr McCanta has been a consultant and a member of the speakers bureau with Biosense Webster/Johnson & Johnson as well as a member of the speakers bureau with Medtronic. All other authors have reported that they have no relationships relevant to the contents of this paper to disclose.

## Appendix

Video 1 Final Lead Position With Integrated Multimodal Imaging (Left Panel) Multimodal imaging of final lead position (blue outline) with three-dimensional electroanatomical map of the Fontan and left pulmonary artery with computed tomography angiogram three-dimensional reconstruction of common atrium and pulmonary veins. Intacardiac echocardiography is positioned in the Fontan to left pulmonary artery junction with the ultrasound sector extending across the cardiac structures. Voltage map with color gradient: red = voltage <0.1 mV; purple = voltage >0.5 mV. (Right Panel) Intracardiac echocardiograpy image of the lead tip positioned in the transpulmonary epicardial space with adherence to the epicardial atrial wall.

Video 2 Rotational Video of Final Lead Location Rotation of three-dimensional electroanatomical map of Fontan and left pulmonary artery with computed tomography angiogram reconstruction of the atrium and pulmonary veins. Voltage map with color gradient: red = voltage <0.1 mV; purple = voltage >0.5 mV.
